# Supplementary material for: Interleukin-6 trans-signaling is a candidate mechanism to drive progression of human DCCs during clinical latency
Source: Nat Commun. 2020 Oct 5;11:4977. doi: 10.1038/s41467-020-18701-4 (PMC7536220; doi:10.1038/s41467-020-18701-4)
Supplement: Supplementary file 1 — Supplementary Information [file 41467_2020_18701_MOESM1_ESM.pdf]

Interleukin-6 trans-signaling is a candidate mechanism to drive progression of human DCCs during clinical latency

Melanie Werner-Klein[1,12]✉, Ana Grujovic[1,9,12], Christoph Irlbeck[1], Milan Obradović[1,10], Martin Hoffmann[2], Huiqin Koerkel-Qu[1], Xin Lu[2], Steffi Treitschke[2], Cäcilia Köstler[2], Catherine Botteron[2], Kathrin Weidele[2], Christian Werno[2], Bernhard Polzer[2], Stefan Kirsch[2], Miodrag Gužvić[1], Jens Warfsmann[2], Kamran Honarnejad[2], Zbigniew Czyz[1], Giancarlo Feliciello[2], Isabell Blochberger[1], Sandra Grunewald[1], Elisabeth Schneider[1], Gundula Haunschild[1], Nina Patwary[1], Severin Guetter[1], Sandra Huber[1], Brigitte Rack[3,11], Nadia Harbeck[3], Stefan Buchholz[4], Petra Rümmele[5,6], Norbert Heine[7], Stefan Rose-John[8] & Christoph A. Klein[1,2] ✉

[1]Experimental Medicine and Therapy Research, University of Regensburg, Regensburg, Germany

[2]Division of Personalized Tumour Therapy, Fraunhofer Institute for Toxicology and Experimental Medicine, Regensburg, 93053, Germany

[3]Department OB&GYN and CCCLMU, Breast center, LMU University Hospital, 80337 Munich, Germany

[4]University Medical Center Regensburg, Clinic of Gynecology and Obstetrics, 93053 Regensburg, Germany

[5]Department of Pathology, University of Regensburg, 93053 Regensburg, Germany

[6]Institute of Pathology, University Hospital, Friedrich-Alexander-University Erlangen-Nürnberg, 91054 Erlangen, Germany

[7]University Center of Plastic-, Aesthetic, Hand- and Reconstructive Surgery, University of Regensburg, Germany

[8]Institute of Biochemistry, Christian-Albrechts-Universität Kiel, Kiel, Germany

[9]Present address: Telexos GmbH, Weilheim, Germany

[10]Present address: Wellmera AG, Basel, Switzerland

[11]Present address: Department of Gynecology, Ulm University Hospital, Ulm, Germany

[12]These authors contributed equally: Melanie Werner-Klein, Ana Grujovic

✉email: melanie.werner-klein@ukr.de, Christoph.klein@ukr.de

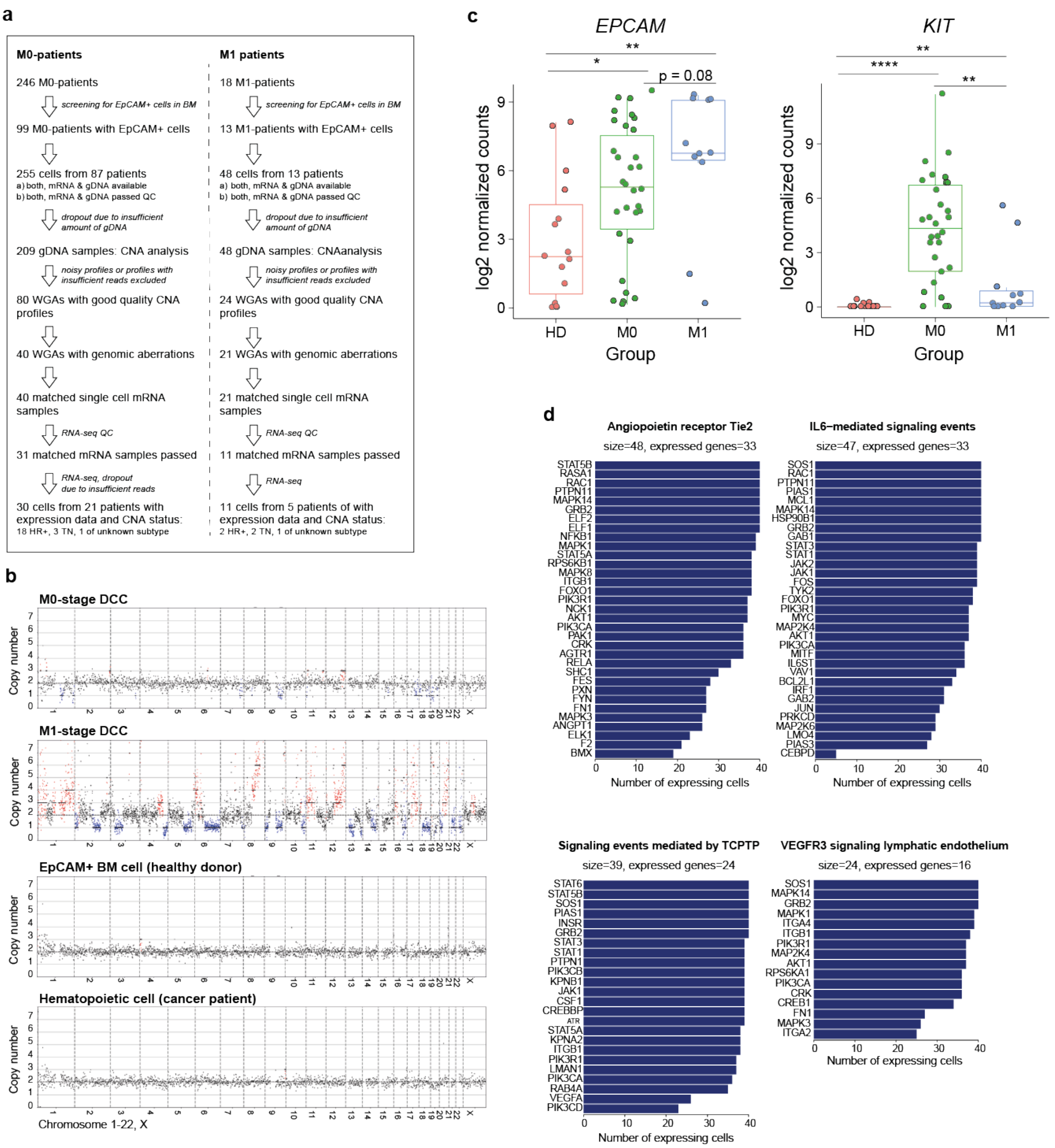

Supplementary Figure 1: Identification and molecular analysis of DCCs from BM of breast cancer patients. a Isolation of EpCAM⁺ DCCs from BM of non-metastasized (M0-stage) and metastasized (M1-stage) breast cancer patients. DNA and RNA were isolated from each cell by WGA and WTA for CNA and RNAseq analysis, respectively. **b** Representative single cell CNA profiles of M0- and M1-stage DCCs and control cells (EpCAM+ cell from BM of a patient without malignant disease or a hematopoietic cell of a cancer patient). **c** Box and whisker plots showing expression of *EPCAM* and *KIT*

in HD (n = 15), M0-stage (n = 30) and M1-stage (n = 11) DCCs with boxes marking the median, lower-quartile and upper-quartile, and lines connecting the extremes. See Supplementary Table 1 for patient/sample-ID allocation. P values according to two-sided Mann-Whitney test; asterisks indicate significance * p <0.05, ** p <0.01, **** p <0.0001; **d** Number of DCCs expressing genes of pathways identified to be enriched in DCCs (see Fig. 3c). Source data are provided as a Source Data file.

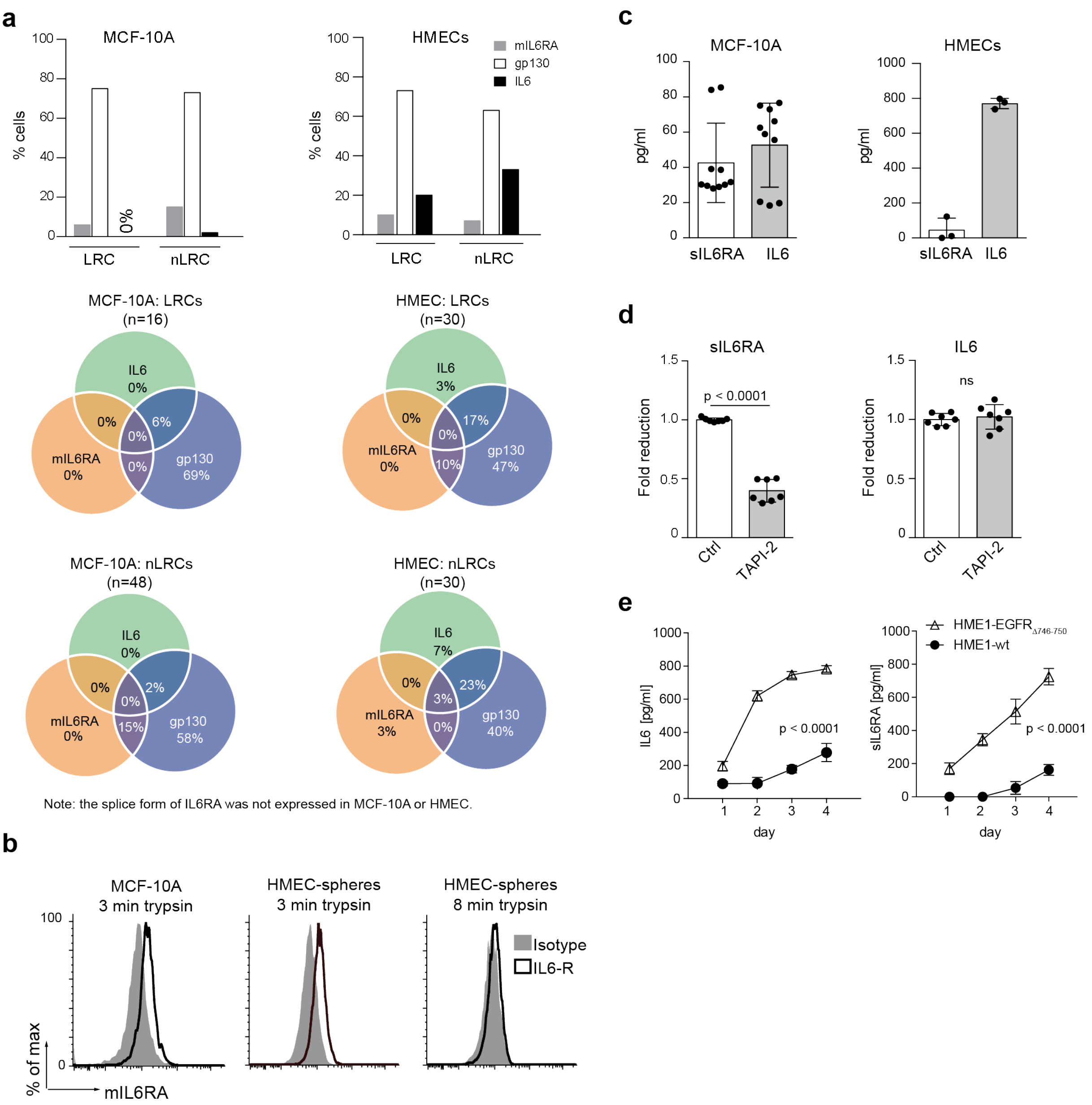

Supplementary Figure 2: Expression of IL6 signaling molecules in MCF-10A, hTERT-HME1 and primary HMECs. **a** Cumulative frequency of IL6, mIL6RA and gp130 mRNA expression in single LRCs or nLRCs from MCF-10A or HMEC-spheres (n = 4 patients). The spliced soluble form of IL6RA was not found to be expressed. Expression of IL6 signaling molecules did not differ significantly between LRCs and nLRCs of MCF-10A or HMECs (LRCs vs. nLRCs for IL6/mIL6Ra/gp130 in MCF-10A or HMECs (Fisher's exact test, p values for all comparisons > 0.05). **b** IL6RA is expressed on the cell surface of MCF-10A cultured under non-sphere conditions and

primary HMEC-spheres. The data is representative of three independently performed experiments. **c** IL6 (n = 10) and soluble IL6RA (n = 10) were measured in the cell culture supernatant of MCF-10A cultured under non-sphere conditions or primary HMEC-spheres (n = 3 patients, each patient analyzed in duplicate). **d** MCF-10A cells were cultured under non-sphere conditions without (n = 7) or with 20 μM TAPI-2 (n = 7), an inhibitor of ADAM-proteases. Protein levels of soluble IL6RA (sILRA) and IL6 in the supernatant were determined by ELISA; n.s. = non significant. **e** IL6 and IL6RA in the supernatant of HME1-wt and isogenic HME1-EGFR$^{\Delta 746\text{-}750}$ cells cultured under non-sphere conditions was determined by ELISA. Cumulative data of two-three experiments, each data point in duplicate. Panel d: two-sided Student's t-test, panel e: linear regression analysis. All error bars correspond to standard deviation (Mean ± SD). See Supplementary Table 1 for patient/sample-ID allocation. Source data are provided as a Source Data file.

a

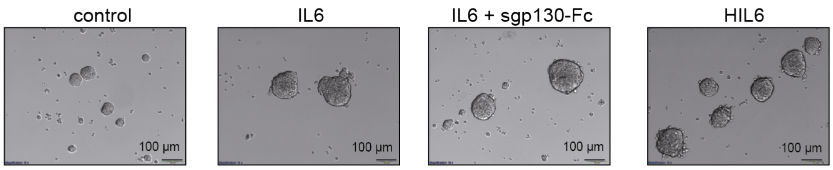

b

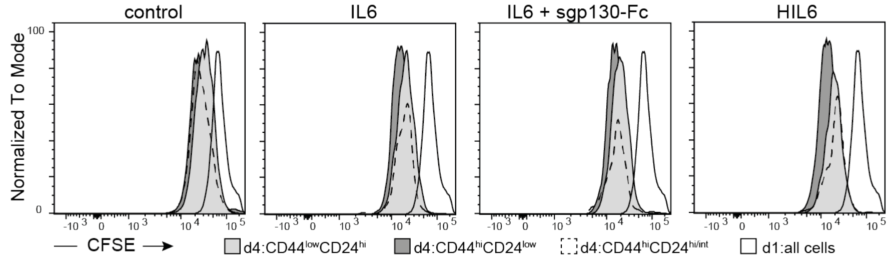

c

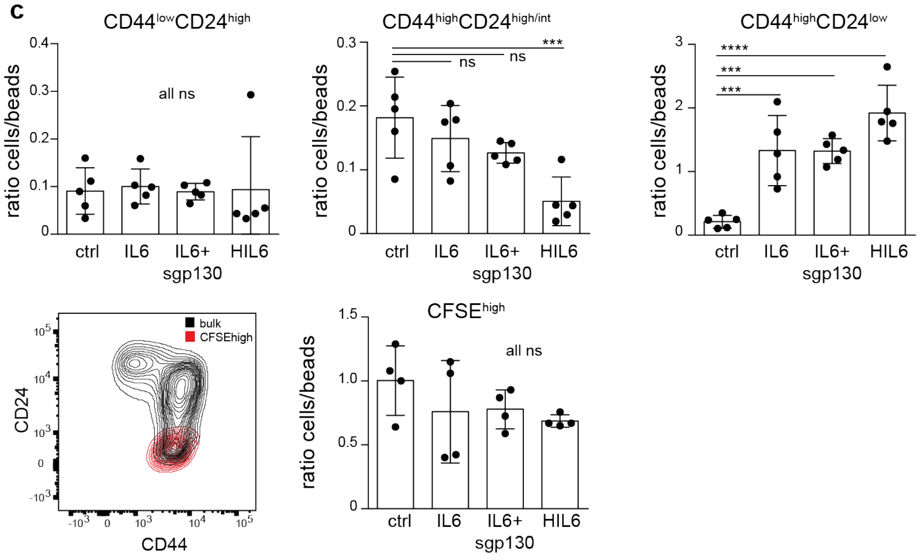

d

	Comparison	Nc cor	Pval nc cor	Num	Pval Num
IL6 classical signaling (IL6+sgp130-Fc)	IL6-induced (12 h) ≙ MatLum→**LumProg**	0.58	***	30	*****
	IL6-induced (12 h) ≙ MaSC→**LumProg**	0.37	*	45	**
	IL6-induced (12 h) ≙ MatLum→MaSC	0.02		42	
	IL6-induced (24 h) ≙ MatLum→**LumProg**	0.27	**	136	*****
	IL6-induced (24 h) ≙ MaSC→LumProg	0.10		285	*****
	IL6-induced (24 h) ≙ MatLum→MaSc	0.05		290	*****
IL6 trans signaling (HIL6)	IL6-induced (12 h) ≙ MatLum→**LumProg**	0.32	***	119	*****
	IL6-induced (12 h) ≙ MaSC→**LumProg**	0.17	**	241	****
	IL6-induced (12 h) ≙ MatLum→MaSC	0.06		257	**
	IL6-induced (24 h) ≙ MatLum→**LumProg**	0.27	****	230	*****
	IL6-induced (24 h) ≙ MaSC→**LumProg**	0.10	*	453	*****
	IL6-induced (24 h) ≙ MatLum→MaSC	0.06		485	*****

MaSC=mammary stem cell enriched LumProg=luminal progenitor MatLum=mature luminal

e

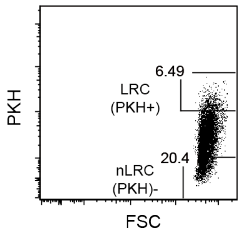

f

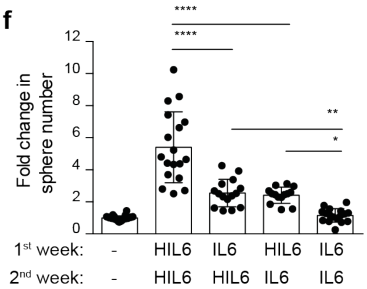

Comparisons	Significance
Ctrl/ctrl vs. IL6/IL6	Ns
Ctrl/ctrl vs. HIL6/IL6	**
Ctrl/ctrl vs. IL6/HIL6	***
Ctrl/ctrl vs. HIL6/HIL6	****
IL6/IL6 vs. HIL6/IL6	*
IL6/IL6 vs. IL6/HIL6	**
IL6/IL6 vs. HIL6/HIL6	****
HIL6/IL6 vs. IL6/HIL6	Ns
HIL6/IL6 vs. HIL6/HIL6	****
IL6/HIL6 vs. HIL6/HIL6	****

Supplementary Figure 3: IL6 trans-signaling converts non-stem cells into stem-like cells. a MCF-10A spheres cultured without or with IL6, IL6 plus sgp130-Fc or with HIL6. **b** CFSE-labeled MCF-10A cells were cultured as spheres with or without activators (IL6, HIL6) and inhibitors of classical (an anti-IL6 antibody) and trans-signaling (sgp130-Fc). CFSE-dilution in $CD44^{high}CD24^{low}$, $CD44^{high}CD24^{high}$ and $CD44^{low}CD24^{high/intermediate}$ cells was determined by flow cytometry at day 4. The CFSE-fluorescence intensity of all cells at day one is included as reference. Data are representative for three 3 independently performed experiments. **c** The absolute number of $CD44^{high}CD24^{low}$, $CD44^{high}CD24^{high}$, $CD44^{low}CD24^{high/intermediate}$ cells (upper panel) and LRCs ($CFSE^{high}$, lower panel) was determined as cell/bead ratio at day 4 by flow cytometry (n = 4-5 per group); n.s. = non significant. **d** Fold-change correlation analysis comparing gene expression changes induced by IL6 plus sgp130 (classical signaling) and HIL6 (trans signaling) in MCF-10 A cells at 12 and 24 hrs with the gene expression signatures of luminal progenitor (LumProg), mature luminal (MatLum) and mammary stem cell enriched cells (MaSC) according to the study of Lim et al.[29]; Nc cor: non-centered correlation between fold-changes, Num: number of common differentially expressed genes. **e** nLRCs from primary, PKH26-labelled control mammosphere-cultures were sorted by flow cytometry as PKH^- cells. **f** Primary HMECs were cultured as spheres for two consecutive rounds in the absence (n = 26) or presence of HIL6 and IL6 (HIL6+HIL6, n = 18; IL6+HIL6, n = 15; HIL6+IL6, n = 14; IL6+IL6, n = 17). Results represent pooled analysis of 14 patients, see Supplementary Table 1 for patient/sample-ID allocation. P values in panel c: one-way ANOVA with Dunett's multiple comparisons test (post-hoc); panel d: p values according to two-sided Student's t-distribution for (transformed) Nc cor and hypergeometric testing for Num. panel f: one-way ANOVA with Tukey's multiple comparisons test (post-hoc); comparisons between groups labeled in red are depicted in the bar graph. Asterisks

indicate significance between groups (* $p < 0.05$ to **** $p < 0.0001$). All error bars correspond to standard deviation (Mean ± SD). Source data are provided as a Source Data file.

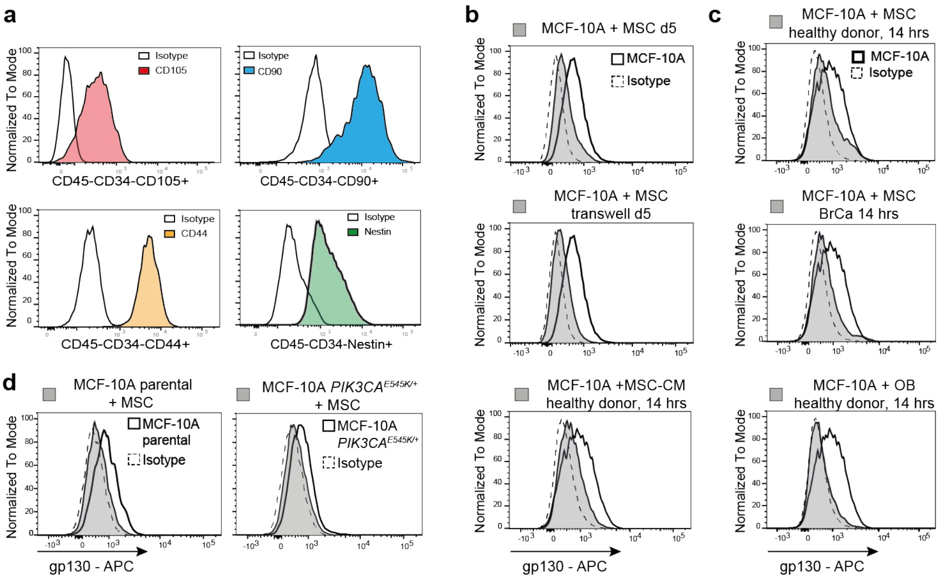

Supplementary Figure 4: Down-regulation of gp130 surface expression by soluble factors of bone marrow stromal cells. a MSCs isolated from bone marrow biopsies of patients without known malignant disease or patients with non-metastasized breast cancer were $CD45^{-}CD34^{-}CD105^{+}CD90^{+}CD44^{+}Nestin^{+}$. **b** gp130 surface expression of MCF-10A cells after five days of co-culture with MSCs or MSCs separated by a transwell or after 14 hrs of co-culture with MSC-conditioned medium (MSC-CM). **c** gp130 surface expression of MCF-10A cells after 14 hours of co-culture with MSCs or OBs from a healthy donor or breast cancer patient. **d** gp130 surface expression on isogenic MCF-10A cells without (MCF-10A parental) or with activating *PIK3CA*$^{E545K/+}$ mutation cultured with MSCs for 5 days. Panel b, c, d: grey filled histograms indicate MCF-10A, MCF-7, or the isogenic cells MCF-10A parental and MCF-10A *PIK3CA*$^{E545K/+}$ cells co-cultured with MSCs, OBs, MSC-CM or MSC separated by a transwell. Histograms with a thick black line indicate MCF-10A or the isogenic cells MCF-10A parental and MCF-10A *PIK3CA*$^{E545K/+}$ cells cultured alone and dashed histograms isotype control staining for gp130.

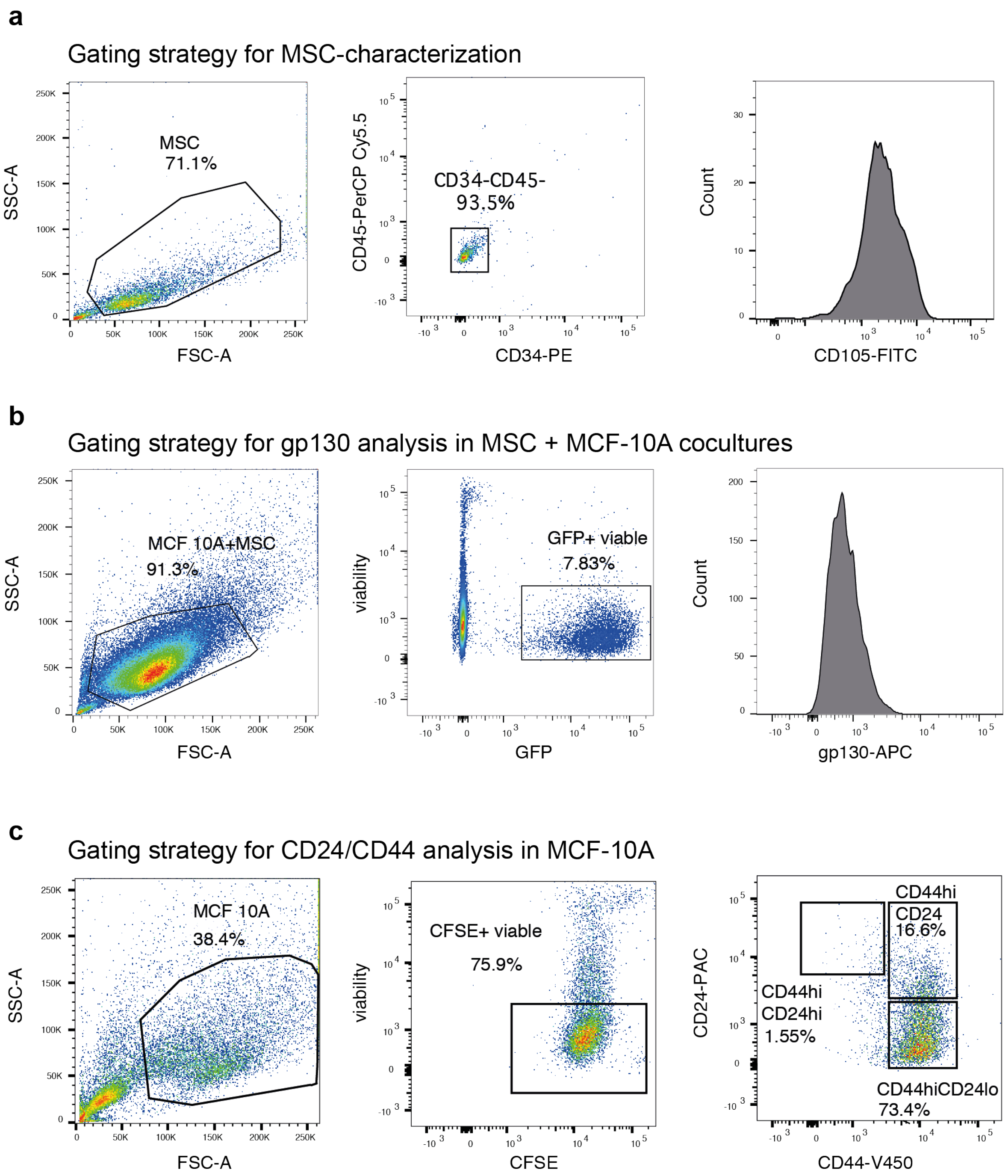

Supplementary Figure 5: Gating strategies for flow cytometric analysis. a MSC-characterization; **b** gp130 analysis in MCF-10A-GFP cells co-cultured with MSCs, OBs or HUVECs; **c** CD24/CD44 analysis in CFSE-labeled MCF-10A cells.
