# Supplementary material for: Interleukin-6 trans-signaling is a candidate mechanism to drive progression of human DCCs during clinical latency
Source: Nat Commun. 2020 Oct 5;11:4977. doi: 10.1038/s41467-020-18701-4 (PMC7536220; doi:10.1038/s41467-020-18701-4)
Supplement: Supplementary file 3 — Description of Additional Supplementary Files [file 41467_2020_18701_MOESM3_ESM.docx]

**Description of Supplementary Files**

**File Name: Supplementary Data 1**

**Description:** Information on patient/sample-ID allocation.

**File Name: Supplementary Data 2**

**Description:** Log2-fold change in gene expression between LRCs, QSCs and nLRCs.

**File Name: Supplementary Data 3**

**Description:** Information on Probe-ID/Gene symbol allocation for microarray analysis.

**File Name: Supplementary Data 4**

**Description:** Information on expressed/non-expressed genes in M0- and M1-stage DCCs.

**File Name: Supplementary Data 5**

**Description:** Gene-members expressed in DCCs and shared among the pathways: "IL6-mediated 136 signaling events", "TCPTP" “VEGF-VEGFR3” and “Angiopoietin-Tie2 receptor”.

**File Name: Supplementary Data 6**

**Description:** Expression fold changes of IL6-induced gene expression in MCF 10A cells at 12 and 140 24 hrs.

**File Name: Supplementary Data 7**

**Description:** Comparative application of statistical tests.

**File Name: Supplementary Data 8**

**Description:** List of all primers used in this study.
