# Supplementary material for: Interleukin-6 trans-signaling is a candidate mechanism to drive progression of human DCCs during clinical latency
Source: Nat Commun. 2020 Oct 5;11:4977. doi: 10.1038/s41467-020-18701-4 (PMC7536220; doi:10.1038/s41467-020-18701-4)
Supplement: Supplementary file 12 — Reporting Summary [file 41467_2020_18701_MOESM12_ESM.pdf]

nature research

Corresponding author(s): Melanie Werner-Klein, Christoph Klein

Reporting Summary

Nature Research wishes to improve the reproducibility of the work that we publish. This form provides structure for consistency and transparency in reporting. For further information on Nature Research policies, see Authors & Referees and the Editorial Policy Checklist.

Statistical parameters

When statistical analyses are reported, confirm that the following items are present in the relevant location (e.g. figure legend, table legend, main text, or Methods section).

n/a	Confirmed	
☐	☒	The exact sample size (*n*) for each experimental group/condition, given as a discrete number and unit of measurement
☐	☒	An indication of whether measurements were taken from distinct samples or whether the same sample was measured repeatedly
☐	☒	The statistical test(s) used AND whether they are one- or two-sided<br>*Only common tests should be described solely by name; describe more complex techniques in the Methods section.*
☐	☒	A description of all covariates tested
☐	☒	A description of any assumptions or corrections, such as tests of normality and adjustment for multiple comparisons
☐	☒	A full description of the statistics including central tendency (e.g. means) or other basic estimates (e.g. regression coefficient) AND variation (e.g. standard deviation) or associated estimates of uncertainty (e.g. confidence intervals)
☐	☒	For null hypothesis testing, the test statistic (e.g. *F*, *t*, *r*) with confidence intervals, effect sizes, degrees of freedom and *P* value noted<br>*Give P values as exact values whenever suitable.*
☒	☐	For Bayesian analysis, information on the choice of priors and Markov chain Monte Carlo settings
☒	☐	For hierarchical and complex designs, identification of the appropriate level for tests and full reporting of outcomes
☐	☒	Estimates of effect sizes (e.g. Cohen's *d*, Pearson's *r*), indicating how they were calculated
☐	☒	Clearly defined error bars<br>*State explicitly what error bars represent (e.g. SD, SE, CI)*

Our web collection on statistics for biologists may be useful.

Software and code

Policy information about availability of computer code

Data collection	Microarray fluorescence readouts were obtained by GenePixPro 7 (Molecular Devices, Germany).
Data analysis	Bioinformatic data analysis was performed using the statistical software R (version 3.5.1 or 3.6.0) with the following R- and bioconductor packages: Rtsne (version 0.15), limma (version 3.36.5), enrichR (version 2.1), rcompanion (version 2.3.7), scater (version 1.12.2), batchelor (version 1.0.1). For read QC we used BBDuk (version 38.69), BioBloom Tools (version 2.0.13), FastQC (version 0.11.8), MultiQC (version 1.8) and for read counting featureCounts (version 1.6.4). Also, NCBI BLAST (version 2.9.0) and STAR (version 2.5.1b) were used for alignment.<br><br>Statistical analysis was performed using the GraphPad Prism 6.0 software (GraphPad Software, Inc., USA). Differences in mean values between groups were analyzed by Student's t-test, Mann-Whitney test or one-way ANOVA followed by post-hoc statistical testing, where appropriate. Time dependencies were analyzed by regression analysis (F-test). Independence in contingency tables was assessed by Fisher's exact test. All tests were realized two-sided. A P value of less than 0.05 was considered statistically significant.<br>Mammosphere counting was performed using Harmony high-content analysis software (Version 4.8, Perkin Elmer, Germany). TissueFAX based cytometric quantification was performed using HistoQuest (6.0.1.130, TissueGnostics, Austria). For FACS, FACS DIVA 5.03 (BD Biosciences, Germany) and FloJo 8.8.6, 10.1 or 10.5.3 (Treestar, USA) was used. Western Blot were analyzed using Image Lab (6.1, Bio-Rad, USA).

For manuscripts utilizing custom algorithms or software that are central to the research but not yet described in published literature, software must be made available to editors/reviewers upon request. We strongly encourage code deposition in a community repository (e.g. GitHub). See the Nature Research guidelines for submitting code & software for further information.

April 2018

Data

Policy information about availability of data

All manuscripts must include a data availability statement. This statement should provide the following information, where applicable:

- Accession codes, unique identifiers, or web links for publicly available datasets
- A list of figures that have associated raw data
- A description of any restrictions on data availability

The microarray (Fig. 2c, 5c) and RNAseq (Fig. 3b, Supplementary Fig. 1c) data have been deposited in the European Genome-phenome Archive under the accession code EGAS00001004597. The source data underlying Figures 1-7 and Supplementary Figures 1-4 are provided as a Source Data file. All the other data supporting the findings of this study are available within the article and its supplementary information files and from the corresponding author upon reasonable request. A reporting summary for this article is available as a Supplementary Information file.

Field-specific reporting

Please select the best fit for your research. If you are not sure, read the appropriate sections before making your selection.

[x] Life sciences   [ ] Behavioural & social sciences   [ ] Ecological, evolutionary & environmental sciences

For a reference copy of the document with all sections, see nature.com/authors/policies/ReportingSummary-flat.pdf

Life sciences study design

All studies must disclose on these points even when the disclosure is negative.

Sample size	Experiments were performed using sample size as indicated throughout the text, figure legends, and methods section.
Data exclusions	No data were excluded from analyses.
Replication	Experiment with cell line cells were repeated at least twice. Replication was successful. Experiments with human samples were repeated for at least with 3 independent patient samples.
Randomization	Bone marrow samples were isolated from unselected, consecutive patients
Blinding	Bioinformaticians were blinded for group allocation

Reporting for specific materials, systems and methods

Materials & experimental systems

n/a	Involved in the study	
[ ]	[x]	Unique biological materials
[ ]	[x]	Antibodies
[ ]	[x]	Eukaryotic cell lines
[x]	[ ]	Palaeontology
[ ]	[x]	Animals and other organisms
[ ]	[x]	Human research participants

Methods

n/a	Involved in the study	
[x]	[ ]	ChIP-seq
[ ]	[x]	Flow cytometry
[x]	[ ]	MRI-based neuroimaging

Unique biological materials

Policy information about availability of materials

Obtaining unique materials	The study comprises patient samples as unique materials. Patient samples underlie legal and ethical restrictions provided by the German federal law and the local ethics committee.

Antibodies

Antibodies used	Flowcytometry: anti-human CD24-APC (ML5), anti-human CD34-PE (581), anti-human CD44-V450 (G44-26), anti-human CD45-

	FITC, APC or PerCP-Cy5.5 (HI30), anti-human CD90 Alexa Flour 700 (5E10), anti-human CD105-FITC (43A3), anti-human CD130-APC (2E1B02), anti-human Nestin-PE (10C2), biotinylated anti-human IL6R (UV4), isotype control mouse IgG2a-APC (MOPC-21), isotype control mouse IgG2b-V450 (MOPC-21), isotype control IgG1-biotin (MOPC-21) (all purchased from BioLegend, Germany) and anti-human EpCAM (HEA-125, Miltenyi-Biotech, Germany). Antibodies were used at the indicated concentration provided by the manufacturers. Individual lot numbers were not recorderd during the study.<br>Western Blot: anti-phospho-STAT3Tyr705 (clone D3A7, 1:2000), anti-phospho-AKTSer473 (clone D9E, 1:2000), anti-phospho-ERK1/2Thr202/Tyr204 (clone E10, 1:2000), anti-STAT3 (clone 124H6, 1:1000), anti-AKT (clone 40D4, 1:2000) and anti-ERK1/2 (clone 137F5, 1:1000). As loading control an anti-α-Tubulin antibody (Sigma-Aldrich, USA, clone DM1A; 1:5000) was used. This was followed by incubation with horseradish peroxidase (HRP)-conjugated goat anti-rabbit IgGs or goat anti-mouse IgGs (both Sigma-Aldrich, USA; 1:10000). Individual Lotnumbers were not recorded during this study.
Validation	Only commercially available antibodies were used that were tested by the manufacturer. In addition, antibodies were verified using positive and negative controls.

Eukaryotic cell lines

Policy information about cell lines

Cell line source(s)	MCF 10A (ATCC); MCF 10A-parental and MCF 10A PIK3CA E545K/+ (Horizon); hTERT-HME1 cell lines (Alberto Bardelli, Italy); C3H10T1/2 (M. Wicha, University of Michigan, USA), MDA-MB-231 (CLS); MCF-7 cells (DSMZ); E4ORF1-HUVECs (Cyrus Ghajar, USA)
Authentication	MCF 10A cell lines , hTERT-HME1 cell lines, MDA-MB-231 and MCF-7 cell lines were authenticated using the GenePrint 10 System (Promega) according to the manufacturer's instructions. C3H10T1/2 and E4ORF1-HUVECs were not authenticated.
Mycoplasma contamination	All cell lines were mycoplasma-tested and found to be negative.
Commonly misidentified lines<br>(See ICLAC register)	No commonly misidentified cell lines were used in this study.

Animals and other organisms

Policy information about studies involving animals; ARRIVE guidelines recommended for reporting animal research

Laboratory animals	Male and female NOD.Cg-Prkdcscid IL2rgtmWjl/Sz (also termed NSG) or NOD.Cg-Prkdcscid mice were purchased from the Jackson Laboratory USA and maintained under specific-pathogen free conditions, with acidified water and food ad libitum in the research animal facilities of the University of Regensburg, Germany. All approved experimental animal procedures were conducted to german federal and state regulations (government of Upper Palatinate, 54-2531.1-10/07, 54-2532.1-34/11; 54-2532.1-17/11, 54-2532.1-1/12, 54-2532.4-7/12). Mice were used at 4-8 weeks of age.
Wild animals	The study did not involve wild animals.
Field-collected samples	The study did not involve samples collected in the field.

Human research participants

Policy information about studies involving human research participants

Population characteristics	Human non-cancerous mammary tissue was obtained from female patients undergoing reduction mammoplasty surgeries at the University Center of Plastic-, Aesthetic, Hand- and Reconstructive Surgery, University of Regensburg, Germany after informed, written consent of patients was obtained (ethics vote number 07/043, ethics committee of the University Regensburg). After verification of the non-cancerous origin of the tissue by a pathologist, mammary glands were dissociated and primary human mammary epithelial cells (HMECs) isolated.<br>Human disseminated cancer cells were obtained from BM-aspirates of breast or prostate cancer patients without and with distant metastases. EpCAM+ cells were obtained from bone marrow of patients without known malignant disease undergoing hip replacement surgery. Human mesenchymal stem cells were obtained from BM-aspirates of breast cancer patients or healthy donors. Written informed consent of cancer and control patients was obtained and the ethics committee of the University of Regensburg (ethics vote number 07/79) approved BM-sampling and analysis of isolated cells.<br>Enrichment and detection of CTCs was performed within the SUCCESS (EUDRA-CT number 2005-000490-21) and DETECT (EUDRA-CT number 2010-024238-46) 68 studies using the CellSearch® system 69. Written informed consent for CTC analysis and characterization was obtained for all patients included. All experiments conformed to the principles set out in the WMA Declaration of Helsinki and were approved by the ethical committees responsible for the corresponding studies (Universities of Munich, Dusseldorf, Tuebingen, and Ulm). Isolation and molecular analysis of CTCs was approved by the ethics committee of Regensburg (ethics vote number 07/079).
Recruitment	According the inclusion criteria (e.g. cancer type), consecutive patients were asked for bone marrow sampling.

Flow Cytometry

Plots

Confirm that:

- [x] The axis labels state the marker and fluorochrome used (e.g. CD4-FITC).
- [x] The axis scales are clearly visible. Include numbers along axes only for bottom left plot of group (a 'group' is an analysis of identical markers).
- [x] All plots are contour plots with outliers or pseudocolor plots.
- [x] A numerical value for number of cells or percentage (with statistics) is provided.

Methodology

Sample preparation	Spheres or adherent cells were trypsinized with trypsin/EDTA (Pan-Biotech, Germany) for 3 min, if not stated otherwise. MSC monocultures and co-cultures of MCF10A-GFP cells with MSCs, OBs and HUVECs were harvested by trypsin/EDTA (Pan-Biotech, Germany) for 5 min and using cell-scrapers. To reduce non-specific binding single cell suspensions were incubated for 5 min at 4° C with PBS/10% AB-serum (Bio-Rad, Germany), subsequently stained with fluorescence-labeled or biotinylated antibodies for 15 min at 4°C and washed once with PBS/2% FCS/0.01% NaN3. In case of biotinylated primary antibodies, PE-labeled streptavidin (Dianova, Germany) was used as secondary staining reagent. Cells were stained using the following antibodies: anti-human CD24-APC (ML5), anti-human CD34-PE (581), anti-human CD44-V450 (G44-26), anti-human CD45-FITC, APC or PerCP-Cy5.5 (HI30), anti-human CD90 Alexa Flour 700 (5E10), anti-human CD105-FITC (43A3), anti-human CD130-APC (2E1B02), anti-human Nestin-PE (10C2), biotinylated anti-human IL6R (UV4), isotype control mouse IgG2a-APC (MOPC-21), isotype control mouse IgG2b-V450 (MOPC-21), isotype control IgG1-biotin (MOPC-21) (all purchased from BioLegend, Germany) and anti-human EpCAM (HEA-125, Miltenyi-Biotech, Germany). Viability dye eFlour 780 (ebioscience, Germany) was used for live/dead cell discrimination.
Instrument	Cells were analyzed on a LSR II machine equipped with FACS DIVA 5.03 software (BD Bioscience, Germany). Sorting of PKH26-labeled LRC and nLRCs was performed with a FACSAria cell sorter (BD Bioscience, Germany).
Software	Data was analyzed with FloJo 8.8.6, 10.1 or 10.5.3 (Treestar, USA).A).
Cell population abundance	Sorting purity was checked on one post-sort population. Purity of sorted cells was >95%.
Gating strategy	For all flowcytometric experiments the following gating strategy was used. Debris was gated out based on FSC-A/SSC-A. GFP+ or CFSE+ live cells were gated based on a plot for CFSE/GFP vs viability dye eFlour 780. Then the markers of interest (CD24,CD44,CD105,CD34,CD45, gp130, CD90, Nestin, IL6RA) were analysed.

- [x] Tick this box to confirm that a figure exemplifying the gating strategy is provided in the Supplementary Information.
